# Is exercise a senolytic medicine? A systematic review

**DOI:** 10.1111/acel.13294

**Published:** 2020-12-30

**Authors:** Xiang‐Ke Chen, Zhen‐Ni Yi, Gordon Tin‐Chun Wong, Kazi Md. Mahmudul Hasan, Joseph Shiu‐Kwong Kwan, Alvin Chun‐Hang Ma, Raymond Chuen‐Chung Chang

**Affiliations:** ^1^ Laboratory of Neurodegenerative Diseases School of Biomedical Sciences LKS Faculty of Medicine The University of Hong Kong Hong Kong China; ^2^ Department of Health Technology and Informatics Hong Kong Polytechnic University Hong Kong China; ^3^ Department of Anaesthesiology LKS Faculty of Medicine The University of Hong Kong Hong Kong China; ^4^ Department of Brain Sciences Imperial College London London UK; ^5^ State Key Laboratory of Brain and Cognitive Sciences The University of Hong Kong Hong Kong China

**Keywords:** cellular senescence, exercise, senescent cells, senolytic medicine, senolytics

## Abstract

Cellular senescence, a state of irreversible growth arrest triggered by various stressors, engages in a category of pathological processes, whereby senescent cells accumulate in mitotic tissues. Senolytics as novel medicine against aging and various diseases through the elimination of senescent cells has emerged rapidly in recent years. Exercise is a potent anti‐aging and anti‐chronic disease medicine, which has shown the capacity to lower the markers of cellular senescence over the past decade. However, whether exercise is a senolytic medicine for aging and various diseases remains unclear. Here, we have conducted a systematic review of the published literature studying the senolytic effects of exercise or physical activity on senescent cells under various states in both human and animal models. Exercise can reduce the markers of senescent cells in healthy humans, while it lowered the markers of senescent cells in obese but not healthy animals. The discrepancy between human and animal studies may be due to the relatively small volume of research and the variations in markers of senescent cells, types of cells/tissues, and health conditions. These findings suggest that exercise has senolytic properties under certain conditions, which warrant further investigations.

## Graphical abstract

Cellular senescent upregulates certain markers such as p16INK4a, p21Cip1, SA‐beta‐Gal, and/or SASP, which can also be shown in accelerated aging animals. This review discusses by using human and experimental laboratory animal studies to show how exercise attenuates all these senescent markers and serves as senolytic medicine.

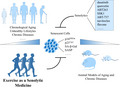

## 1 INTRODUCTION

Aging, especially when coupled with unhealthy lifestyles, is the leading contributor to most physical dysfunctions and chronic diseases in humans, whereby senescent cells defined as division‐arrested normal cells progressively accumulate in tissues (Childs et al., 2015; Collado et al., 2007). While gradual cellular senescence throughout the whole aging process was identified by Hayflick and Moorhead in 1961, to date, rare anti‐aging and age‐associated disease medicine has been developed targeting these biological aging processes at the cellular level. Moreover, unhealthy lifestyles, such as physical inactivity, unhealthy diets, and cigarette smoking, have been found to be associated with the accumulation of senescent cells in humans independent of chronological age (Liu et al., 2009; Song et al., 2010; Tchkonia et al., 2010). Importantly, cellular senescence also participates in other vital biological processes, not only aging but tumor suppression, tumor promotion, and tissue repair (Rodier & Campisi, 2011). Therefore, while cellular senescence is a promising therapeutic target of multiple diseases, especially aging‐related diseases and cancer, the consequences and safety of up‐ or downregulating these complex biological processes should be taken into consideration.

Senescent cells are the hallmark and therapeutic target of cellular senescence involved in a wide range of biological processes, including tumor suppression, embryonic development, wound healing and tissue repair, and aging (Van Deursen, 2014). Although senescent cells are detrimental to the body during aging and can lead to chronic diseases, such as obesity, diabetes, and sarcopenia, they also suppress cancer and fibrosis (Muñoz‐Espín & Serrano, 2014). Under the complex functions, there are two major senescent pathways: p53/p21^Cip1^ and p16^INK4a^/RB; and both p21^Cip1^ and p16^INK4a^ are the key markers of senescent cells (Ben‐Porath & Weinberg, 2005). Moreover, other markers of senescent cells have been reported, including senescence‐associated heterochromatic foci (SAHFs), ARF, DNA segments with chromatin alterations reinforcing senescence (DNA‐SCARS), senescence‐associated beta‐galactosidase (SA‐β‐Gal), shorter telomere length, lower proliferation, and senescence‐associated secretory phenotype (SASP) (He & Sharpless, 2017). Exploration of these senescent markers as targets might contribute to the development and testing of anti‐senescent cell therapeutic strategies.

Senolytics is a new class of medicines that target senescent cells, which has emerged rapidly in the past few years (Kirkland et al., 2017). Preclinical studies on rodents have been applied to explore the potential targets of senescent cells and the preliminary effects of senolytic medicine in vivo. In 2011, an INK‐ATTAC transgenic mouse with a BubR1 progeroid background was first established and showed restored aging‐associated dysfunctions following ablation of senescent cells by AP20187 (Baker et al., 2011). Moreover, another transgenic line, p16‐3MR, was generated, where senescent cells can also be eliminated via ganciclovir and further mitigated the post‐traumatic osteoarthritis and age‐related intervertebral disk degeneration (Jeon et al., 2017; Patil et al., 2019). In addition to transgenic mice, senolytic drugs, including dasatinib and quercetin, ABT263, and SSK1, also showed the therapeutic effects on senescent cells and alleviated the radiation and age‐related symptoms and pathology (Cai et al., 2020; Chang et al., 2016; Zhu et al., 2015). A combined treatment of senolytic medicines in transgenic mice also presented the therapeutic effects on obesity‐induced metabolic dysfunction and fibrotic pulmonary disease (Palmer et al., 2019; Schafer et al., 2017). Strikingly, two small clinical trials on senolytic treatments with dasatinib and quercetin were completed last year and reported therapeutic effects for patients with diabetic kidney disease (*N* = 9) and idiopathic pulmonary fibrosis (*N* = 14) (Hickson et al., 2019; Justice et al., 2019). More recently, other novel senolytic agents such as ABT‐737, navitoclax, flavone, fisetin, A1331852, and A1155463 have been developed; ongoing research will determine their effects against various diseases in the future (Yosef et al., 2016; Zhu et al., 2015, 2017).

Physical exercise is widely recognized as a safe, effective, and cost‐effective “medicine” for a broad range of age‐related diseases. Moreover, a lack of exercise is a major contributing factor to accelerated aging and age‐associated chronic conditions, including cancer, obesity, and cardiovascular diseases (Booth et al., 2011). World Health Organization (WHO) thus encourages adults aged 18–64 years to engage in over 150 min of moderate‐intensity physical activity each week to reduce the risk of these chronic conditions. Unfortunately, 23% of men and 32% of women worldwide failed to meet this recommendation according to data from WHO, (Guthold et al., 2018). Therefore, a clearer delineation of anti‐aging and anti‐disease effects and underlying mechanisms of exercise is needed. While the accumulation of senescent cells has been identified as the mechanism of aging and multiple diseases for decades, senolytics targeting senescent cells has just been developed in recent years. In addition, exercise has shown its capacity to lower the marker of senescent cells over the past decade. In the current systematic review of all available literature, we explored the potential senolytic effects of exercise in both human and animal models under healthy or disease states. We aimed to improve the understanding of the cellular senescence‐based mechanisms underlying exercise as anti‐aging medicine. This may impel people to engage in more exercise and lead to the development of more precise “exercise prescriptions” and “exercise mimetics” for the aging population and patients with various age‐related diseases (Li & Laher, 2015).

## 2 RESULTS

### 2.1 Study characteristics

**FIGURE 1:**
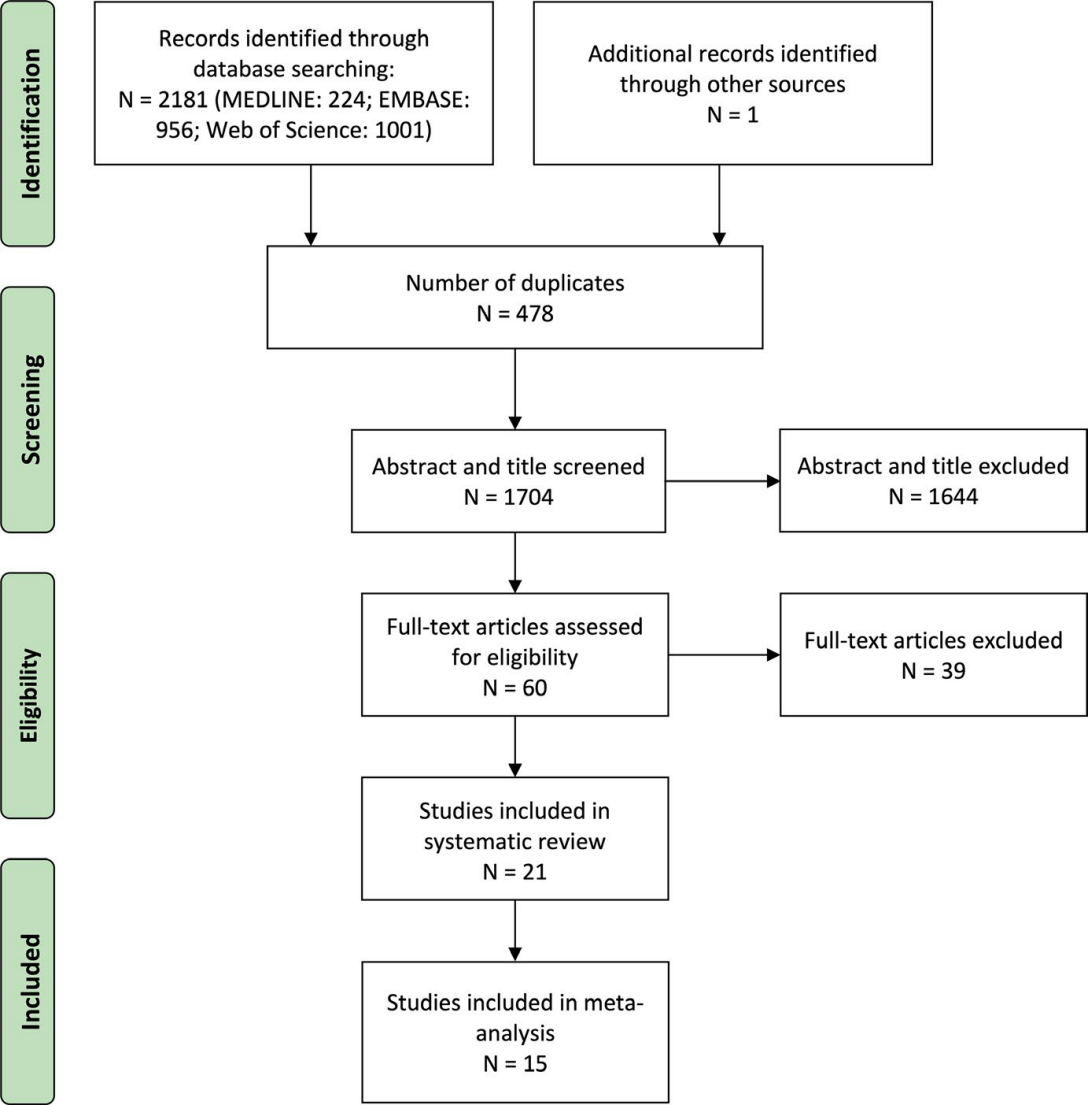
PRISMA flowchart of search strategy

The systematic search in the database and manually searching the reference list yielded 2182 articles. After the exclusion of duplicates, 1704 articles were screened for the abstract and title. Of these, 60 articles were further screened for full text and finally included 21 articles in this review, which contained eight human studies, twelve animal studies, and one study included both human and animal experiments (Figure 1). The total number of included participants in human studies was 535 (Table 1). Participants, including sedentary or active volunteers, athletes, and patients, aged 18–81 years were recruited. Four out of nine were cohort studies, while three of them were intervention studies.

**TABLE 1:** Senolytic effects of exercise on senescent cells in human studies

Author (year)	Participant	Exercise	PA Levels/Training protocol	Organ or cell	Marker of SCs	Senolytics
*Habitual PA (Untrained)*						
Liu et al. (2009)	Healthy participants (18–80 years); *n* = 170	Habitual PA	0–1900 min/month (*n* = 170). Comparison between <= 240 and >240 min/month	T lymphocyte	p16^INK4a^↓	Yes
Tsygankov et al. (2009)^a^	Healthy participants (18–80 years ); *n* = 170	Habitual PA	0–1900 min/month (*n* = 170). Comparison between <= 240 and >240 min/month	T lymphocyte	p16^INK4a^↓	Yes
Song et al. (2010)	Healthy participants (18–80 years); *n* = 104	Habitual PA	>= 0 and <2000 min/month (*n* = 104)	T lymphocyte	p16^INK4a^↓	Yes
Pustavoitau et al. (2016)	Older (56–81 years) patients undergoing coronary artery bypass surgery; *n* = 47	Habitual PA	>= 0 and <4000 min/month (*n* = 47). Comparison between <= 240 and >240 min/month	T lymphocyte	p16^INK4a^→	No
*Chronic exercise training*						
Werner et al. (2009)	Healthy middle‐aged marathon runners or ﻿triathletes (51.1 ± 1.6 years) and sedentary control (50.9 ± 1.6 years); *n* = 46	Exercise training (running)	Trained: 80 ± 7.5 km/week for 35 ± 2.7 years; control: < 1 hr per week for 1 years	Mononuclear cells	p16^INK4a^↓	Yes
Rossman et al. (2017)	Healthy trained older participants (59 ± 1 years, LTPA: 83 ± 9 MET hr/week) and sedentary control (62 ± 1 years, LTPA: 32 ± 6 MET hr/week) control; *n* = 25	Exercise training	Trained: >= 45 min/day, 5 days/week for 5 years; control: <30 min/day, 2 days/week for 2 years	Vascular endothelial cell	p16^INK4a^↓ & p21^Cip1^↓	Yes
Justice et al. (2018)	Older overweight/obese women (67–77 years); *n* = 8	Resistance training (machine)	3 sets of 10 repetitions at 70% 1RM per day, 3 day/week for 5 month	Adipose tissue	p16^INK4a^↓	Yes
*Acute exercise bout*						
Yang et al. (2018)	Healthy recreationally active (>= twice a week for >3 years) college student (22.5 ± 1.7 years); *n* = 12	Resistance exercise (squat)	6 sets of 12 repetitions at 70% of 1 RM	Endothelial progenitor cells	p16^INK4a^↓	Yes
Wu et al. (2019)	Healthy recreationally active men (21 ± 0.2 years); *n* = 12	Bicycle ergometer	1 h on bike ergometer at 70% power output	Muscle	SA‐β‐gal→	No

Abbreviations: ↓ deceased and →unchanged compared with control (non‐exercise);LTPA, leisure time physical activity; MET, metabolic equivalent; PA, physical activity; RM, repetition maximum; SA‐β‐gal, senescence‐associated beta‐galactosidase; SCs, senescent cells.

^a^ The same cohort as (Liu et al., 2009) used for development of mathematical model that links p16^INK4a^ with aging.

Overall, most human studies used p16^INK4a^ and T lymphocyte as markers of senescent cells and targeted tissue or cell, respectively. For physical exercise, habitual physical activity (via questionnaire) was determined in most human studies, while resistance training and an acute bout of moderate‐high intensity cycling were applied as the exercise intervention. On the other hand, mice (nine studies) and rat (five studies) were utilized as animal models to investigate the senolytic effects of exercise on senescent cells (Table 2). Wild‐type and spontaneous or accelerated aging animals were used in most studies. Overall, p16^INK4a^, p21^Cip1^, and/or SA‐β‐Gal were applied as markers of senescent cells in various tissues or cells. For chronic exercise training, long‐term running, including both wheel/voluntary and treadmill/forced running, was reported in most animal studies, while swimming was also applied as the exercise intervention in three studies.

**TABLE 2:** Senolytic effects of exercise on senescent cells in animal studies

Author (year)	Animal model	Exercise	Exercise protocol	Organ or tissue	Marker of SCs	Senolytics
*Chronic exercise training*						
Werner et al. (2008)	Mice (8‐week‐old); *n* = 8–12^a^; WT	Wheel running (voluntary)	5100 ± 800 m/24 h for 21 days	Heart	p16^INK4a^↓	Yes
Werner et al. (2009)	Mice (8‐week‐old); *n* = 6–8; WT	Wheel running (voluntary)	4280 ± 670 m/24 h for 21 days	MNC & Vessel	p16^INK4a^↓	Yes
Kröller‐Schön et al. (2012)	Mice (8‐week‐old); *n* = 6; WT	Wheel running (voluntary)	4336 ± 842 m/24 h for 8 weeks	Endothelium	p16^INK4a^↓	Yes
Huang et al. (2013)	Rat (7‐week‐old); *n* = 5–6; Accelerated aging	Swimming	60 min/time, 5 times/week for 12 weeks	Liver	p21^Cip1^↓& SA‐β‐gal↑	Opposite & Yes
Schafer et al. (2016)	Mice (8‐month‐old); *n* = 6–7; Obesity	Wheel running (voluntary)	3291 ± 1166 m/24 h for 16 weeks	Adipose tissue, liver, aorta, skeletal muscle, pancreas, kidney, heart,	p16^INK4a^↓→, p21^Cip1^↓→& SA‐β‐gal↓→	Yes & No
Zhang et al. (2016)	Rat (20‐month‐old); *n* = n.a.; Spontaneous Aging	Treadmill running	30 min/day at 13 m/min, 5 days/week for 4–8 weeks	Tendons	SA‐β‐gal↓	Yes
Fan et al. (2017)	Rat (4‐month‐old); *n* = 7; Accelerated aging	Swimming	45 min/day, 5 day/week for 6 weeks	Muscle	SA‐β‐gal↓	Yes
Yoon et al. (2019)	Mice (9‐week‐old/19‐month‐old); *n* = 5; Spontaneous Aging	Treadmill running	30 min, twice per day, 5 days/week for 4 weeks	Muscle	p16^INK4a^↓→& p21^Cip1^↓→	Yes & No
Wong et al. (2019)	Mice (3–5‐month‐old/23‐24‐month‐old); *n* = 5–7; Spontaneous Aging	Treadmill running	33d at 10 m/min and 5 day at 8 m/min	Epidermal and dermal cell	p16^INK4a^→& p21^Cip1^→	No
Liu et al. (2019)	Rat (8–10‐week‐old); *n* = 3; WT	Swimming	60 min/day for 5 week with sinker weight	Hippocampus cell	p21^Cip1^↑& SA‐β‐gal↑	Opposite
Jang et al. (2019)	Mice (10‐week‐old); *n* = 3; Obesity	Treadmill running	12‐week running, 1 h/day, 5 day/week with increased speed (12–14–15 m/min)	Hippocampus cell	p16^INK4a^↓, p21^Cip1^↓, SA‐β‐gal↓ and lipofuscin↓	Yes
Bao et al. (2020)	Mice (19‐month‐old); *n* = 3; Spontaneous Aging	Rotatable treadmill running	15–60 min/day, 5 day/week for 6 weeks with increased time and speed (3.2–4.8 m/min)	Kidney	SA‐β‐gal↓	Yes
*Acute exercise bout*						
Saito et al. (2020)	Mice (13‐week‐old); *n* = 3–5; WT	Treadmill running	−20° downhill treadmill for 30 min at 17 m/min	Fibro‐adipogenic progenitors	p16^INK4a^→& p21^Cip1^↑	Opposite & No

^a^ Per group; WT, wild‐type; SCs: senescent cells; SA‐β‐gal: senescence‐associated beta‐galactosidase; ↑ increased, ↓deceased and → unchanged compared with control (non‐exercise).

### 2.2 Qualitative description of study findings

Of all 21 articles, 16 articles reported senolytic effects of exercise on the markers of senescent cells characterized by declined levels of specific molecular machinery (p16^INK4a^, p21^Cip1^, and SA‐β‐Gal), while five and three articles reported no and opposite effects of exercise on senescent cells, respectively. Interestingly, exercise showed distinct effects on different senescent markers (e.g., p16^INK4a^ and p21^Cip1^) in one study.

For human studies (Table 1), the level of habitual physical activity is negatively associated with the level of p16^INK4a^ in immune cells of healthy untrained participants aged 18–80 years (Liu et al., 2009; Song et al., 2010; Tsygankov et al., 2009), but not in older coronary bypass patients aged 56–81 years (Pustavoitau et al., 2016). In addition, chronic exercise training reduced the level of p16^INK4a^ or p21^Cip1^ in mononuclear cells or vascular endothelial cells of healthy middle‐age athletes (marathon runners or triathletes) and healthy older trained participants compared to sedentary controls (Rossman et al., 2017; Werner et al., 2009). On the other hand, chronic resistance training reduced the level of p16^INK4a^ in aged and overweight/obese women (4 out of 8 participants underwent caloric restriction during training were excluded) (Justice et al., 2018). A single bout of resistance training acutely lowered the level of p16^INK4a^ in healthy recreationally active college students (Yang et al., 2018), but an acute bout of moderate‐high intensity cycling failed to reduce the level of SA‐β‐Gal in the muscle of healthy recreationally active young men (Wu et al., 2019). Collectively, physically active people have lower levels of p16^INK4a^‐positive senescent cells than sedentary people, while resistance training contributed to a lower level of p16^INK4a^.

For animal studies (Table 2), prolonged voluntary wheel running decreased the level of p16^INK4a^ in various organs/tissues or cells in young, old, or obese mice/rat, including heart, vessel, endothelium, and adipose tissue (Kröller‐Schön et al., 2012; Schafer et al., 2016; Werner et al., 2008, 2009), while it remained unchanged in some other organs and tissues, such as kidney and pancreas. Moreover, long‐term forced treadmill running showed contradictory effects on the markers of senescent cells, including p16^INK4a^, p21^Cip1^, and SA‐β‐Gal (Bao et al., 2020; Jang et al., 2019; Wong et al., 2019; Yoon et al., 2019; Zhang et al., 2016), although four out of five studies showed senolytic effects of exercise in specific tissues under obesity or aging conditions. Conversely, studies on an acute bout of downhill running and prolonged swimming reported an increased level of p21^Cip1^ and SA‐β‐Gal in fibro/adipogenic progenitors (Saito et al., 2020), and liver and brain (Huang et al., 2013; Liu et al., 2019), respectively, while another swimming study indicated a reduced level of SA‐β‐Gal in the muscle (Fan et al., 2017). In summary, 10 out of 13 animal studies showed a senolytic effect of exercise, but this effect was influenced by the form and dosage of exercise, type of senescent tissue or cells, and healthy or aging/disease conditions.

### 2.3 Meta‐analysis

Overall, 16 out of 21 articles were included in the meta‐analysis. However, two studies were excluded because of a lack of essential data (Wu et al., 2019; Zhang et al., 2016), one studies were excluded because of less than two data with similar outcomes and design (Saito et al., 2020), one study was excluded because the data were taken from cohort reported in another study (Tsygankov et al., 2009), and one study was excluded because of the fact that exercise was not an independent factor (Yang et al., 2018).

**FIGURE 2:**
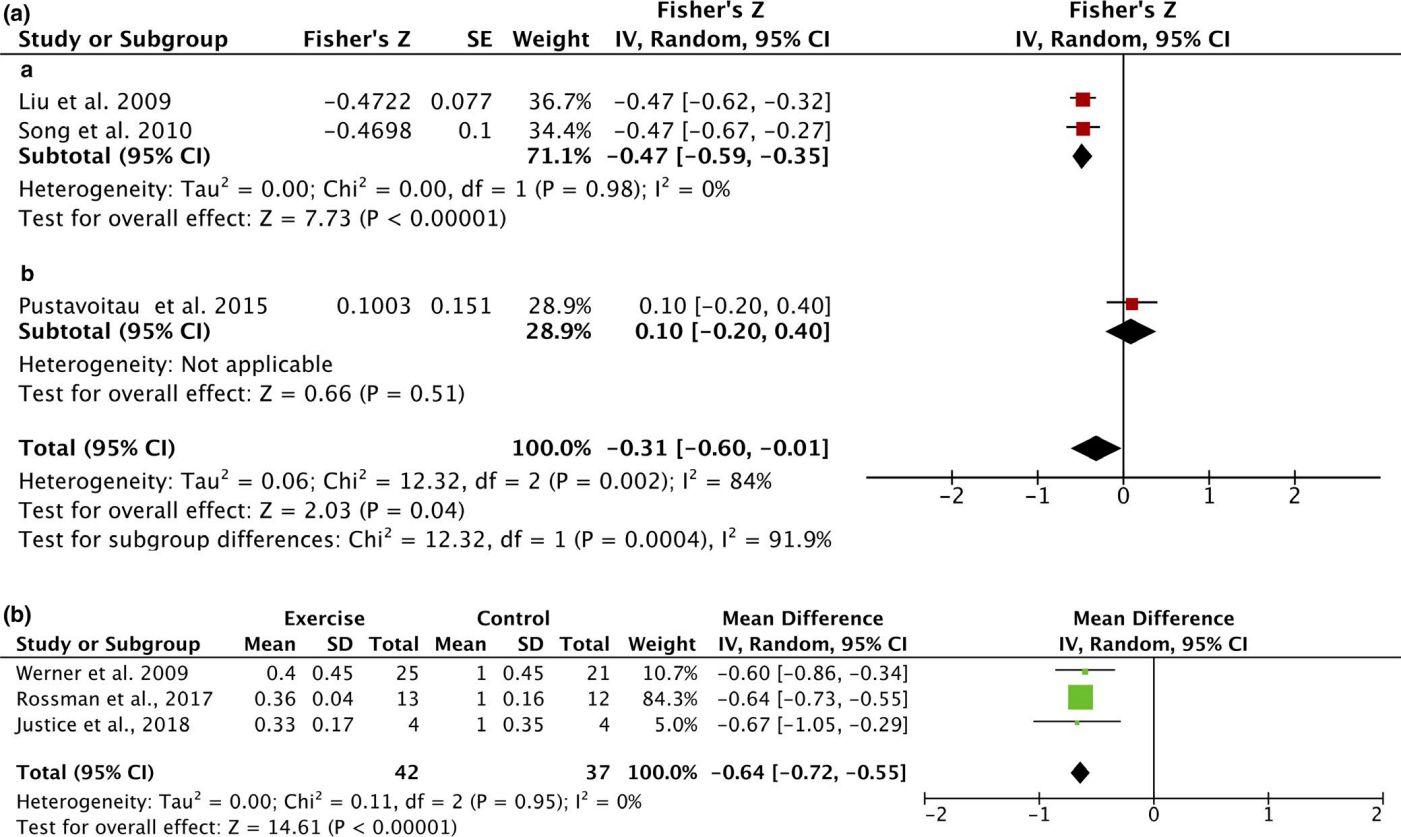
Forest plots of the meta‐analysis for the effect of exercise on the level of p16^INK4a^ in human studies. A: association of habitual exercise and the level of p16^INK4a^ in T lymphocyte with subgroup analysis. (a) healthy participants; (b) patients. B: the effect of exercise training on p16^INK4a^

For cross‐sectional studies in humans, a negative correlation between habitual physical activity and the level of p16^INK4a^ in T lymphocytes (*r* = −0.30, 95% CI = −0.54, 0.01, *I* ^2^ = 84%, *p* = 0.04), subgroup by healthy participants (*r* = −0.30, 95% CI = −0.53, −0.34, *I* ^2^ = 0%, *p* < 0.001) and patients (one article) were identified (Figure 2a). Moreover, exercise training significantly reduced the level of p16^INK4a^ in humans (−64%, 95% CI = −72%, −55%, *I* ^2^ = 0%, *p* < 0.001) (Figure 2b). While both habitual physical activity and exercise training studies supported the senolytic effects of exercise on p16^INK4a^‐positive senescent cells, more studies on various exercise and markers of senescent cells in different populations are still required.

**FIGURE 3:**
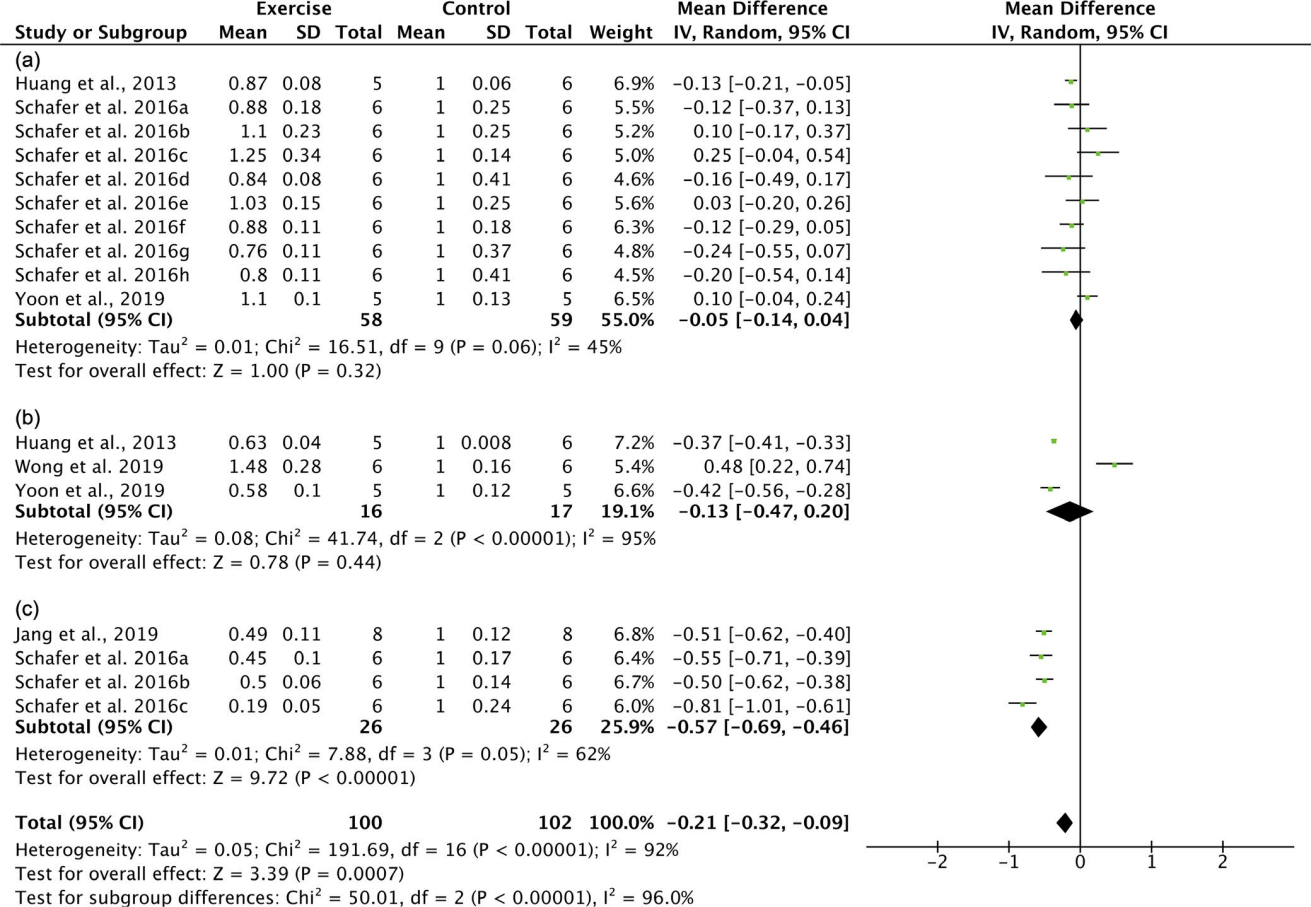
Forest plots of the subgroup meta‐analysis for effect of exercise on the level of p21^Cip1^ in healthy and young animals. Subgroup by healthy states: (a) health; (b) aging; (c) high‐fat‐diet (HFD)‐induced obesity. Schafer et al. (2016) a‐h (various organs and tissues): a, visceral fat; b, subcutaneous fat; c, liver; d, gastrocnemius (muscle); e, pancreas; f, kidney; g, heart; h, aorta

On the other hand, diverse effects of exercise on the level of senescent cells were observed in animal studies. p16^INK4a^ and p21^Cip1^ were conventional markers of senescent cells used in previous animal studies. Specifically, no significant effects of exercise on p16^INK4a^ were found in animal studies with high heterogeneity (−11%, 95% CI = −31%, 10%, *I* ^2^ = 99%, *p* = 0.32) (Figure S1). To identify the source of the high heterogeneity, studies were subgrouped by tissues, including heart, vessel, muscle, fat, skin, brain, liver, pancreas, and kidney. Of these, a senolytic effect of exercise was observed in vessel with a lower heterogeneity (−56%, 95% CI = −72%, −39%, *I* ^2^ = 73%, *p* < 0.001), while no significant effect of exercise was found in muscle (42%, 95% CI = −51%, 136%, *I* ^2^ = 59%, *p* = 0.37). For p21^Cip1^, a significant senolytic effect of exercise was observed in animal studies, though a high heterogeneity was observed (21%, 95% CI = −32%, −9%, *I* ^2^ = 92%, *p* < 0.001) (Figure 3). To identify the source of the high heterogeneity, studies were subgrouped by healthy states, including healthy and young, aged, and HFD‐induced obesity. Strikingly, no effect of exercise on p21^Cip1^ was found in a healthy state (−5%, 95% CI = −14%, 4%, *I* ^2^ = 45%, *p* = 0.32). In contrast, a significant senolytic effect of exercise on senescent cells was found in obese (−57%, 95% CI = −69%, −46%, *I* ^2^ = 62%, *p* < 0.001) but not aged animals (*p* = 0.44). SA‐β‐gal, another marker of senescent cells, was also examined in the animal studies; however, high heterogeneity was observed together with the senolytic effects of exercise, which cannot be reduced by subgroup (−40%, 95% CI = −64%, −16%, *I* ^2^ = 99%, *p* < 0.001) (Figure S2). In summary, senolytic effects of exercise were observed in the vessel (p16^INK4a^) and obese animals (p21^Cip1^), but not in the muscle (p16^INK4a^) or healthy animals (p21^Cip1^). In this context, the senolytic effects of exercise remain unclear, especially in various organs or tissues and states. More studies with similar outcomes and designs are needed due to the high level of heterogeneity observed in these studies.

### 2.4 Study quality

The quality scores of each study included in this systematic review are shown in the supplemental materials. For human cross‐sectional studies, the score of Newcastle‐Ottawa quality assessment ranged from 6 (satisfactory studies) to 8 (good studies) (Table S2). Moreover, AUB KQ1 risk of bias assessment of human intervention studies reported a low risk of random sequence generation (80%), allocation concealment (60%), incomplete outcome data (100%), selective outcome reporting (100%), and other bias (100%), but not blinding of patients and personnel (20%) and blinding of outcome assessment (20%) (Table S3). On the other hand, the median number of CAMARADES checklist score for animal studies was 4 ranged from 3 to 6 (Table S4). Although the quality assessment of animal studies was provided, the risk of bias remained unclear in these studies due to the lack of sufficient details for assessment that has long been identified as the limitation of animal research.

### 2.5 Publication bias

Publication bias was examined for outcomes with over nine data/studies by the asymmetry of funnel plots. No major bias was detected in animal studies on p16^INK4a^ (Figure S3) or p21^Cip1^ (Figure S4), while some asymmetry was observed in the funnel plots, but this was unlikely to be due to publication bias.

## 3 DISCUSSION

Our systematic review and meta‐analysis were the first to investigate the senolytic effects of exercise on senescent cells in both human and animal studies. Despite the limited number of studies, the evidence on the senolytic effect of exercise on senescent cells in humans and animals appears convincing. A higher level of habitual physical activity and exercise training decreased the level of p16^INK4a^ in humans. In addition, high heterogeneity was observed in animal studies, probably due to study design and sample heterogeneity rather than a real heterogeneity in results. A further subgroup analysis revealed that exercise reduced the level of p21^Cip1^ in various organs/tissues under high‐fat‐diet (HFD)‐induced obesity. In contrast, no senolytic effect of exercise on the level of p21^Cip1^ was found in the healthy state. The evidence for other outcomes and conditions is less clear. Thus, more human and animal studies investigating the senolytic effects of exercise on senescent cells are required.

Cellular senescence serves pleiotropic roles in aging, chronic diseases, and cancer (Van Deursen, 2014). In addition to advancing age, unhealthy lifestyles, such as physical inactivity, unhealthy diets, and cigarette smoking, also contribute to the accumulation of senescent cells in the body, especially during aging, and subsequent accelerated aging and chronic diseases (Liu et al., 2009; Song et al., 2010). In this context, the elimination of senescent cells (senolytics) is a potential therapy for aging and chronic diseases, such as obesity, type 2 diabetes, and atherosclerosis (He & Sharpless, 2017). Senolytic medicine has been developed in recent years and some of them are currently undergoing clinical trials, which may require years to be clinically available (Hickson et al., 2019; Justice et al., 2019). Our systematic review and meta‐analysis found evidence that exercise has senolytic properties in healthy humans, where a higher level of habitual physical activity or physically active (Liu et al., 2009; Song et al., 2010) and chronic exercise training (Justice et al., 2018; Rossman et al., 2017; Werner et al., 2009) reduced the level of p16^INK4a^ in various tissues/cells, especially the senescent lymphocytes. These findings supported the hypothesis that exercise can be a senolytic medicine, although there was only very limited evidence demonstrating a negative association between habitual exercise/exercise training and the level of senescent markers. Besides, the optimal or the lowest effective dose of exercise remains unclear (Eijsvogels & Thompson, 2015). Therefore, studies on the different forms (e.g., exercise alone or together with other senolytics such as dasatinib and quercetin) and dosages of exercise on senescent cells in various cells/tissues, populations, and health conditions are urgently needed. Moreover, exercise may have potential anti‐tumor properties (Pedersen et al., 2016), which raises the question of whether exercise is beneficial for cancer patients in the context of senescent cells as an emerging target of cancer (Lee & Schmitt, 2019). Collectively, physical exercise shows promise as a senolytic medicine against aging and multiple diseases, which calls for more preclinical and clinical studies.

On the other hand, animal studies on the senolytic effects of exercise provided more evidence about its effects on various tissues and organs in detail, which cannot be directly investigated in humans. A recent systematic review and meta‐analysis reported that the expression levels of selected markers of senescent cells varied from different tissues during aging (Tuttle et al., 2020). Similarly, we found no significant change of p16^INK4a^ in animal studies with high heterogeneity. However, a significant decline of p16^INK4a^ was detected in the vessels (Kröller‐Schön et al., 2012; Schafer et al., 2016; Werner et al., 2009) but not the muscle (Saito et al., 2020; Schafer et al., 2016; Yoon et al., 2019) after subgroup analysis by tissues. In accordance with a previous study that p16^INK4a^ expressed in various organs and tissues, such as the brain, liver, and spleen, but not in the muscle, during aging (Idda et al., 2020), while senescent cells may be the therapeutic target of cardiovascular diseases (Childs et al., 2018). It suggested that senolytic medicine, including exercise, may only be suitable to target the aging and disease states for certain tissues and organs. The comprehensive cellular senescence‐based effects of exercise on various organs and tissues remain to be investigated; the results of these studies may contribute to the development of more precise exercise therapeutic strategies in the future.

In addition to p16^INK4a^, p21^Cip1^ is another conventional marker of senescent cells. Unlike p16^INK4a^ which is involved in the maintenance of senescent phenotype, p21^Cip1^ is essential for establishing senescence (Stein et al., 1999). Strikingly, we found exercise showed no effect on the level of p21^Cip1^ in healthy and young animals (Huang et al., 2013; Schafer et al., 2016; Yoon et al., 2019). Since cellular senescence serve vital roles in health maintenance and cancer suppression (He & Sharpless, 2017), a lack of significant senolytic effects of exercise on p21^Cip1^ in healthy animals may imply potential safety of using exercise as senolytic medicine, since exercise may only “remove” excess senescent cells induced by pathological stressors. On the contrary, in the HFD‐induced obesity, exercise reduced the level of p21^Cip1^ in obese animals after subgroup analysis by the disease states, including aging and obesity, while more studies are required to understand its effects on senescent cells in aged animals (Jang et al., 2019; Schafer et al., 2016). Cellular senescence is considered to be involved in and serves as the therapeutic target of obesity and metabolic dysfunction (Palmer et al., 2019). However, few disease states have been examined in previous animal studies. Exercise may be a potent and safe senolytic medicine against a specific disease, especially for senescent cells characterized by p21^Cip1^.

While the senolytic effects of exercise have been identified in the current review, the underlying mechanisms are less well understood. Previous studies have reported that exercise regulated DNA damage (Radák et al., 2002), telomere erosion (Puterman et al., 2010), oxidative stress (Gomez‐Cabrera et al., 2008), in various tissues and cells, are the major drivers of cellular senescence. On the other hand, exercise‐induced autophagy (He, Bassik, et al., 2012; He et al., 2012) and apoptosis (Phaneuf & Leeuwenburgh, 2001), which have been shown to elicit regulating effects on cellular senescence (Kang et al., 2015; Vicencio et al., 2008), might also be involved in the senolytic effects of exercise. A clearer picture of mechanisms underlying senolytic effects of exercise may contribute to the discovery and development of “exercise mimetics” or “exercise pills” against senescent cells (Li & Laher, 2015) and encourage people to engage in more exercise to tackle physical inactivity as one of the leading health problems in the world (Blair, 2009; Kohl et al., 2012) and to develop exercise as an effective treatment for specific diseases.

The present systematic review and meta‐analysis provide an up‐to‐date summary of the senolytic effects of exercise on senescent cells based on both human and animal studies. However, there are some limitations that should be mentioned. The major limitation of this study is the limited number of studies included in this review, though the effects of exercise on senescent cells have been studied for over a decade. In addition, the relatively high heterogeneity was observed among studies due to the complexity of research on cellular senescence, such as the types of cells/tissues and markers of senescent cells. Besides, the search strategy was limited to the articles written in English, which may lead to a language bias and a potential loss of studies met other items of inclusion criteria.

The findings of this systematic review and meta‐analysis provided some evidence that exercise may be a senolytic medicine for p16^INK4a^‐positive senescent cells in humans and for p21^Cip1^‐positive senescent cells in obese but not healthy animals. Future studies should examine the optimal form and dosage of exercise, targeted cells/tissues, different disease states, and the underlying cellular mechanisms in humans and animals. A greater understanding of the senolytic effects of exercise can lead to significant clinical and public health impact.

## 4 METHODS

### 4.1 Search strategy and data sources

A systematic search of the literature was conducted on May 20, 2020, in three databases: MEDLINE (OVID), EMBASE (OVID), and Web of Science using the Medical Subject Headings (MeSH) terms “exercise” and “cellular senescence” without date limit (a full list of search items used in systemic search is listed in Table S1). Articles were imported into Mendeley reference management software (Elsevier, USA), and the duplicates were identified and removed. Titles and abstracts of all identified records were independently screened by two reviewers to exclude irrelevant articles. The specific inclusion criteria were as follows: (a) original study; (b) peer‐reviewed article; (c) articles written in English; (d) full text available; (e) animal or human studies; (f) “senescence” or “senescent” means “cellular senescence”; (g) “exercise” means physical exercise. Subsequently, full text of the remaining articles was retrieved and reviewed by the same independent reviewers with the following additional inclusion criteria: (a) markers of senescent cells (p16^INK4a^, p21^Cip1^, SA‐β‐Gal, and lipofuscin) were reported; (b) exercise as a sole intervention/contributing factor; (c) for immunosenescence, articles were included if cellular senescence in immune cells was reported.

### 4.2 Study quality assessment

The quality of included studies was assessed independently by two reviewers using (a) Newcastle‐Ottawa quality assessment scale for cohort studies, (b) AUB KQ1 risk of bias assessment, and (c) collaborative approach to meta‐analysis and review of animal data from experimental studies (CAMARADES) checklist (Anon n.d.) for (a) habitual physical activity studies in humans, (b) exercise training studies in humans, and (c) animal studies, respectively. The information of each article was then summarized after independent assessment and the disagreement was resolved by discussion.

### 4.3 Extraction of data

Data were extracted independently by the same reviewers. Any disagreement was resolved by discussion or a third reviewer. The following study characteristics were extracted onto a pre‐designed data collection form: author, publication year, participant or animal characteristics (e.g., age, health and exercise status, type of animal), sample size, types of exercise, physical activity level/training protocol, types of organ, tissue or cell, markers of senescent cells and their responses to exercise, senolytic effect (yes, no, or opposite). For analysis purposes, means, standard deviation, and sample size were collected for each study. If the data were reported in graphical form, means and standard deviation were extracted using WebPlotDigitizer (Rohatgi, 2011). Studies were excluded if the mean, standard deviation or sample size was not available or equal to 0. If the sample size was provided as a range, we utilized the smallest value. To examine the effects of exercise in different markers of senescent cells, these data were extracted separately. Moreover, to determine whether the senolytic effects of exercise were specific to certain disease state, data from healthy/young samples and disease /old samples were extracted separately as well. Therefore, the total number of data points included in the meta‐analysis was greater than the number of articles.

### 4.4 Data analysis

For all meta‐analyses, review manager (RevMan) version 5.3 (Copenhagen: The Nordic Cochrane Center) was used where at least two studies of similar study design had reported the same outcomes (i.e., markers of senescent cells). For each study, the mean differences based on the changes from control to exercise group, or from baseline to post‐exercise, were calculated and pooled using the random‐effects model. Correlation coefficient (*r*) was used to calculate the effect size after transforming to Fisher's z in meta‐analysis Fisher's z was then transformed back to r for presentation. Heterogeneity was determined using Cochran's chi‐square test and Higgins's *I* ^2^ test while publication bias was explored using funnel plots. To assess the specific senolytic effects of exercise on senescent cells in animal studies, we pre‐defined the following subgroups for analyses: markers of senescent cells, healthy and young/disease or old, and organ/tissue. Sensitivity analysis was performed by removing the lowest quality study among included studies from the analysis.

## CONFLICT OF INTEREST

The authors declare that they have no conflicts of interest.

## AUTHOR CONTRIBUTIONS

XKC, RCC, ACM, and JSK contributed to the conception and design of this study. XKC, ZNY, and KMH contributed to literature searching and eligible study screening. XKC and ZNY contributed to the data extraction and analysis. XKC, ZNY, KMH, GTW, RCC, ACM, and JSK contribute to the manuscript preparation and reviewed the final version.

## Supporting information

Supplementary Material

## Data Availability

We believe this systematic review is exempt under the rule of data availability.
