# Supplementary material for: Is exercise a senolytic medicine? A systematic review
Source: Aging Cell. 2020 Dec 30;20(1):e13294. doi: 10.1111/acel.13294 (PMC7811843; doi:10.1111/acel.13294)
Supplement: Supplementary file 1 — Supplementary Material [file ACEL-20-e13294-s001.docx]

**Supporting information**

**Tables**

**Table S1.** Search terms for articles searching

|  | **Search terms** |
| --- | --- |
| **Physical exercise** | Exercises OR Physical Activity OR Activities, Physical OR Activity, Physical OR Physical Activities OR Exercise, Physical OR Exercises, Physical OR Physical Exercise OR Physical Exercises OR Acute Exercise OR Acute Exercises OR Exercise, Acute OR Exercises, Acute OR Exercise, Isometric OR Exercises, Isometric OR Isometric Exercises OR Isometric Exercise OR Exercise, Aerobic OR Aerobic Exercise OR Aerobic Exercises OR Exercises, Aerobic OR Exercise Training OR Exercise Trainings OR Training, Exercise OR Trainings, Exercise |
| **Senescent cells** | Senescent OR Senescence OR Senescence, Cellular OR Cell Senescence OR Senescence, Cell OR Cell Aging OR Cellular Ageing OR Ageing, Cellular OR Aging, Cell OR Senescence, Replicative OR Cellular Aging OR Aging, Cellular OR Replicative Senescence OR Cell Ageing OR Ageing, Cell OR Senescence-Associated Secretory Phenotype OR Phenotype, Senescence-Associated Secretory OR Secretory Phenotype, Senescence-Associated OR Senescence Associated Secretory Phenotype OR SASP |

**Table S2.** Newcastle-Ottawa quality assessment (adapted version for cross-sectional studies) of human cross-sectional studies

| Study | Selection | Comparability | Outcomes | Total |
| --- | --- | --- | --- | --- |
| Liu et al. (2009) | ******* | ****** | ******* | **8** |
| Tsygankov et al. (2009) | ******* | ****** | ******* | **8** |
| Song et al. (2010) | ******* | ****** | ******* | **8** |
| Pustavoitau et al. (2015) | ****** | ****** | ****** | **6** |

Good Studies: 7-8 points; Satisfactory Studies: 5-6 points; Unsatisfactory Studies: 0 to 4 points

**Table S3.** AUB KQ1 risk of bias assessment of human intervention studies

| **Study** | **Random sequence generation** | **Allocation concealment** | **Blinding of patients and personnel** | **Blinding of**  **outcome assessment** | **Incomplete outcome data** | **Selective outcome reporting** | **Any other bias** |
| --- | --- | --- | --- | --- | --- | --- | --- |
| Werner et al. (2009) | **High** | **Unclear** | **High** | **Unclear** | **Low** | **Low** | **Low** |
| Rossman et al. (2017) | **Low** | **Unclear** | **Unclear** | **Unclear** | **Low** | **Low** | **Low** |
| Justice et al. (2018) | **Low** | **Low** | **High** | **High** | **Low** | **Low** | **Low** |
| Yang et al. (2018) | **Low** | **Low** | **Unclear** | **Unclear** | **Unclear** | **Low** | **Low** |
| Wu et al. (2019) | **Low** | **Low** | **Low** | **Low** | **Low** | **Low** | **Low** |

**Table S4**. Collaborative approach to meta-analysis and review of animal data from experimental studies (CAMARADES) quality assessment of animal studies

| **Study** | **Publication in peer-reviewed journal** | **Statement of control of temperature** | **Randomization of treatment or control** | **Allocation concealment** | **Blinded assessment** | **Avoidance of anesthetics with marked intrinsic properties** | **Use of animals with hypertension or diabetes** | **Sample size calculation** | **Statement of compliance with regulatory requirements** | **Statement regarding possible conflict of interest** | **Total (on 10)** |
| --- | --- | --- | --- | --- | --- | --- | --- | --- | --- | --- | --- |
| Werner et al. (2008) | 🗸 |  | 🗸 |  | 🗸 |  |  |  | 🗸 |  | 4 |
| Werner et al. (2009) | 🗸 |  | 🗸 |  |  |  |  |  | 🗸 | 🗸 | 4 |
| Kröller-Schön et al. (2012) | 🗸 |  |  |  |  |  |  |  | 🗸 | 🗸 | 3 |
| Huang et al. (2013) | 🗸 | 🗸 | 🗸 |  |  |  |  |  | 🗸 | 🗸 | 5 |
| Schafer et al. (2016) | 🗸 |  | 🗸 |  |  |  | 🗸 |  | 🗸 | 🗸 | 5 |
| Zhang et al. (2016) | 🗸 |  |  |  |  | 🗸 |  |  | 🗸 | 🗸 | 4 |
| Fan et al. (2017) | 🗸 | 🗸 | 🗸 |  |  | 🗸 |  |  | 🗸 | 🗸 | 6 |
| Yoon et al. (2019) | 🗸 |  | 🗸 |  |  |  |  |  | 🗸 | 🗸 | 4 |
| Wong et al. (2019) | 🗸 |  | 🗸 |  |  | 🗸 |  |  | 🗸 |  | 4 |
| Liu et al. (2019) | 🗸 |  | 🗸 |  |  |  |  |  | 🗸 |  | 3 |
| Jang et al. (2019) | 🗸 |  | 🗸 |  |  | 🗸 | 🗸 |  | 🗸 | 🗸 | 6 |
| Saito et al. (2020) | 🗸 |  | 🗸 |  |  |  |  | 🗸 | 🗸 | 🗸 | 5 |
| Bao et al. (2020) | 🗸 | 🗸 |  |  |  |  |  |  | 🗸 | 🗸 | 4 |

**Figures**

**Figure S1.** Forest plots of the subgroup meta-analysis for the effect of exercise on p16INK4a-positive senescent cells in animal studies. Subgroup by tissue or cells: (a) heart; (b) vessel; (c) muscle; (d) fat; (e) skin; (f) brain; (g) liver; (h) pancreas; (i) kidney.

**
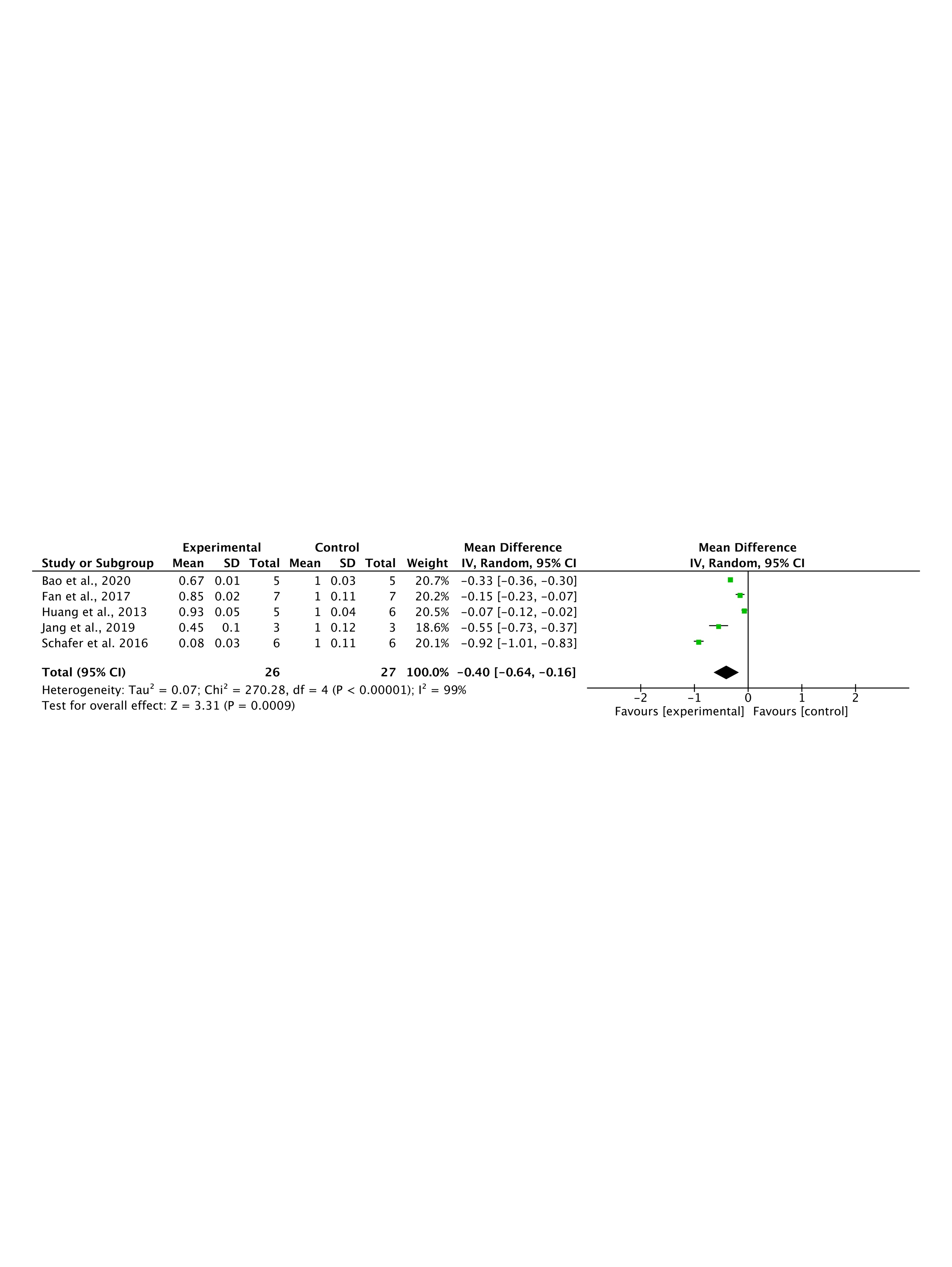
Figure S2**. Forest plots of the subgroup meta-analysis for the effect of exercise on SA-β-Gal positive senescent cells in animal studies.


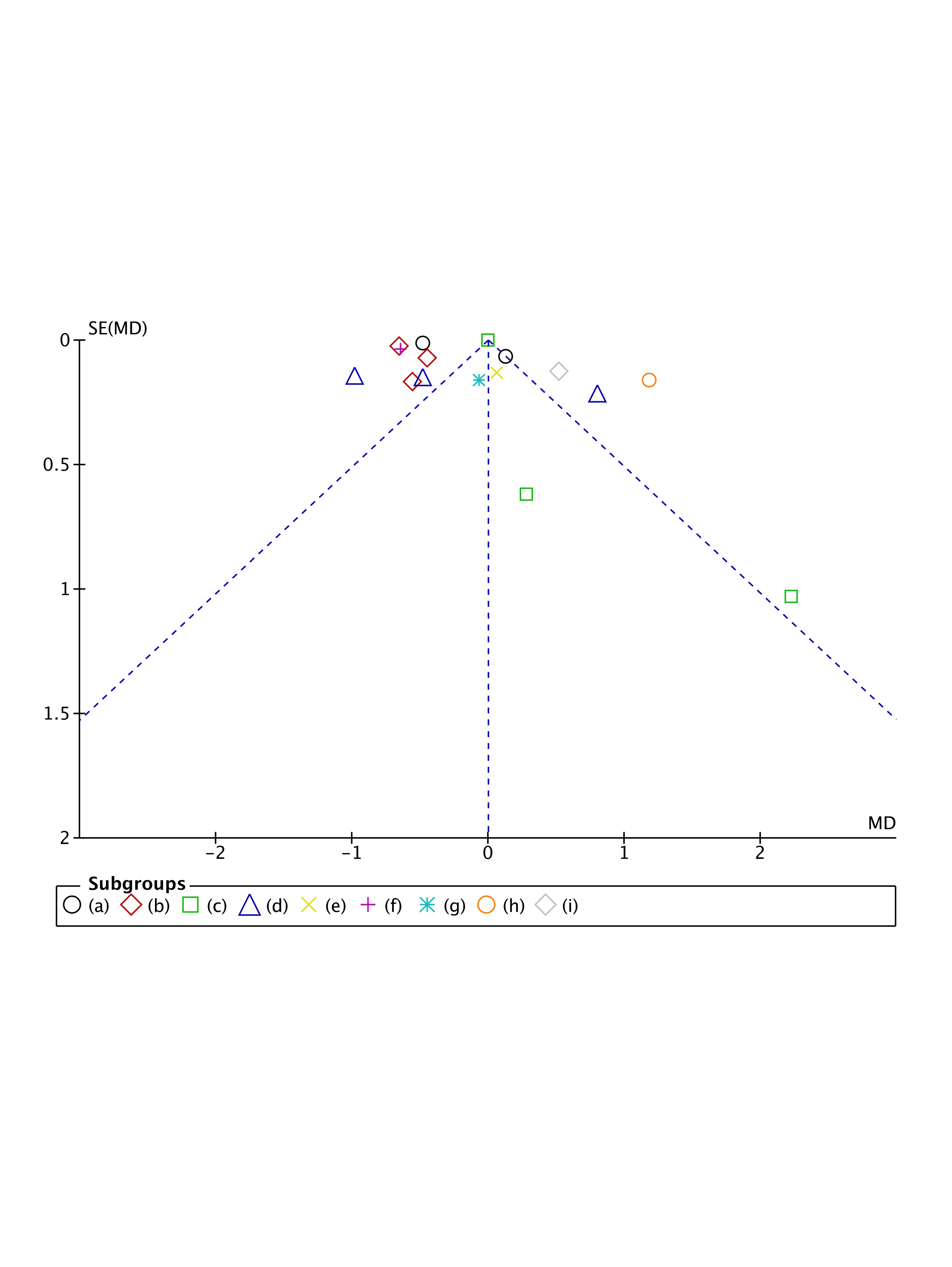


**Figure S3**. Begg's funnel plot for the senolytic effect of exercise on p16^INK4a^

(N=16)

**
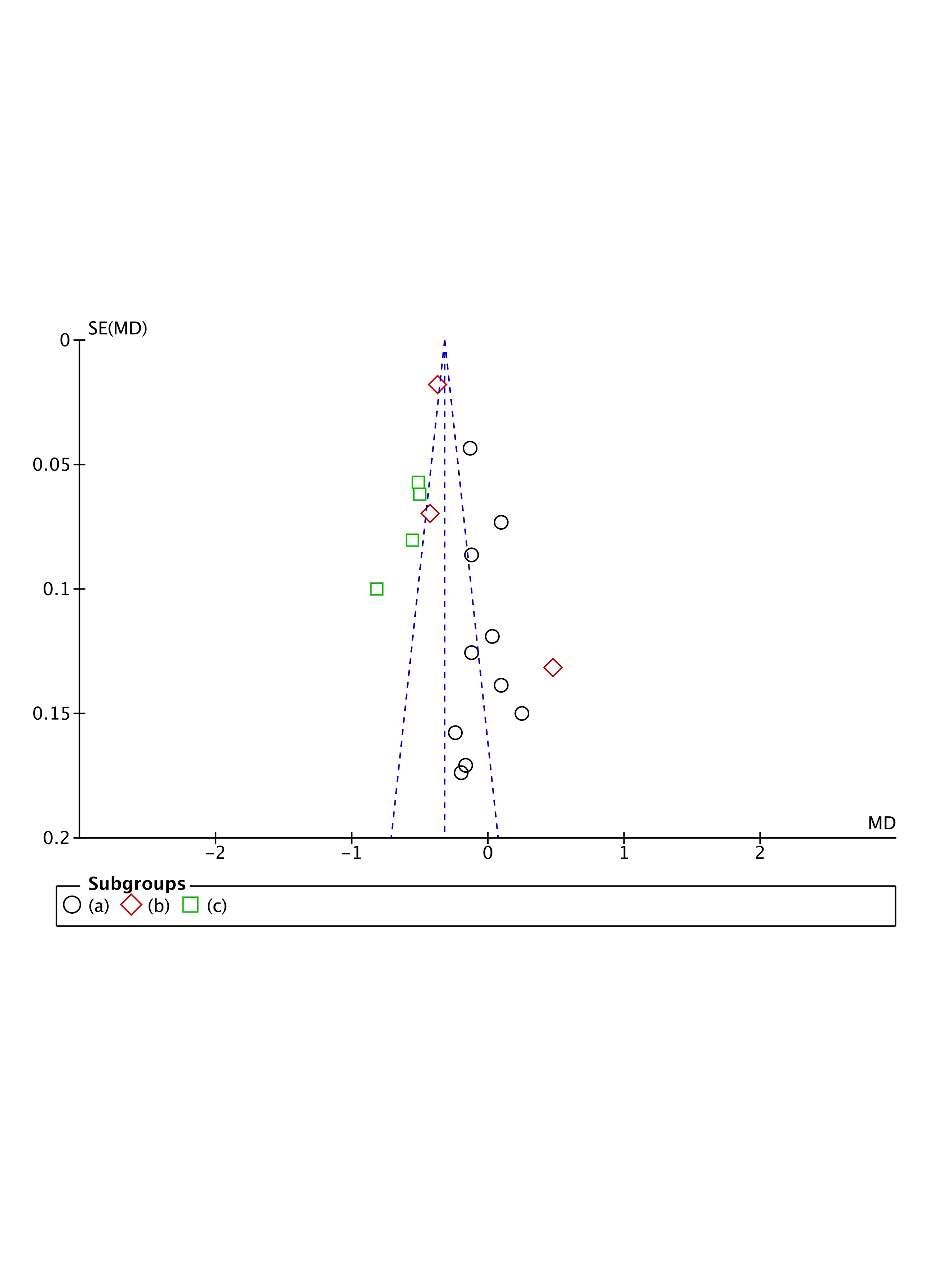
Figure S4**. Begg's funnel plot for the senolytic effect of exercise on p21^Cip1^

(N=17)
